# Development and Validation of a Prognostic Model to Predict Recurrence-Free Survival After Curative Resection for Perihilar Cholangiocarcinoma: A Multicenter Study

**DOI:** 10.3389/fonc.2022.849053

**Published:** 2022-04-21

**Authors:** Zhi-Peng Liu, Wei-Yue Chen, Zi-Ran Wang, Xing-Chao Liu, Hai-Ning Fan, Lei Xu, Yu Pan, Shi-Yun Zhong, Dan Xie, Jie Bai, Yan Jiang, Yan-Qi Zhang, Hai-Su Dai, Zhi-Yu Chen

**Affiliations:** ^1^ Department of Hepatobiliary Surgery, Southwest Hospital, Third Military Medical University (Army Medical University), Chongqing, China; ^2^ Department of Clinical Research Institute, Lishui Hospital of Zhejiang University, Lishui, China; ^3^ Department of General Surgery, 903rd Hospital of People’s Liberation Army, Hangzhou, China; ^4^ Department of Hepatobiliary Surgery, Sichuan Provincial People’s Hospital, Chengdu, China; ^5^ Department of Hepatobiliary Surgery, Affiliated Hospital of Qinghai University, Xining, China; ^6^ Department of Health Statistics, College of Military Preventive Medicine, Third Military Medical University (Army Medical University), Chongqing, China

**Keywords:** perihilar cholangiocarcinoma, prognostic model, recurrence, resection, oncology

## Abstract

**Background:**

Recurrence is the main cause of death in perihilar cholangiocarcinoma (pCCA) patients after surgery. Identifying patients with a high risk of recurrence is important for decision-making regarding neoadjuvant therapy to improve long-term outcomes.

**Aim:**

The objective of this study was to develop and validate a prognostic model to predict recurrence-free survival (RFS) after curative resection of pCCA.

**Methods:**

Patients following curative resection for pCCA from January 2008 to January 2016 were identified from a multicenter database. Using random assignment, 70% of patients were assigned to the training cohort, and the remaining 30% were assigned to the validation cohort. Independent predictors of RFS after curative resection for pCCA were identified and used to construct a prognostic model. The predictive performance of the model was assessed using calibration curves and the C-index.

**Results:**

A total of 341 patients were included. The median overall survival (OS) was 22 months, and the median RFS was 14 months. Independent predictors associated with RFS included lymph node involvement, macrovascular invasion, microvascular invasion, maximum tumor size, tumor differentiation, and carbohydrate antigen 19-9. The model incorporating these factors to predict 1-year RFS demonstrated better calibration and better performance than the 8th American Joint Committee on Cancer (AJCC) staging system in both the training and validation cohorts (C-indexes: 0.723 *vs.* 0.641; 0.743 *vs.* 0.607).

**Conclusions:**

The prognostic model could identify patients at high risk of recurrence for pCCA to inform patients and surgeons, help guide decision-making for postoperative adjuvant therapy, and improve survival.

## Introduction

Cholangiocarcinoma (CCA) is an epithelial tumor with features of cholangiocyte differentiation. It originates from the ductal epithelium of the biliary tree from the canals of Hering to the main bile duct, and although it accounts for only 3% of gastrointestinal tumors, the incidence has gradually increased in the past decade (1, 2). According to the anatomical location, 60%-70% of cholangiocarcinomas are perihilar (3, 4). While curative resection is the recommended treatment for perihilar cholangiocarcinoma (pCCA), the 5-year overall survival (OS) is very poor, at only 25%-35%, and recurrence is the main cause of death (5, 6). Thus, screening out pCCA patients with a high risk of recurrence after curative resection has become a critical step.

At present, the 8th American Joint Committee on Cancer (AJCC) TNM has been proposed to predict oncologic outcomes for patients. However, it lacks accuracy because AJCC staging lacks many prognostic factors (7). With the deepening of the studies, most of the factors related to prognosis after curative pCCA resection have been determined, including tumor differentiation, macro- or microvascular invasion, tumor size, lymph node (LN) status, and serum tumor biomarkers (8–12). For LN status, provided that the number of examined lymph nodes (ELNs) is less than 4, prediction systems may falsely indicate negative LN involvement, which was demonstrated to be an independent risk factor for poor oncologic prognosis of pCCA (13–15). For tumor size, patients with tumor size > 3 cm have a poorer prognosis (16). Moreover, tumor size > 5 cm was also found to be related to poor survival of pCCA (17). Based on these studies, it may be possible to refine the tumor size to more accurately predict the long-term prognosis of pCCA patients. Notably, in the past 5 years, several studies have developed models to predict the long-term prognosis of pCCA, but all of them lack serum tumor biomarkers (18–21). Carbohydrate antigen 19-9 (CA 19-9) is a known serum tumor biomarker that is independently associated with the long-term prognosis of pCCA (22). As a consequence, this study tried to add the above mentioned variables to one prognostic model may further improve the prediction performance of individual patients after curative pCCA resection. Despite that, predicting the long-term oncologic outcomes of individual patients remains challenging. A nomogram is a visual and simple prognostic model system that can predict the long-term outcome of individual patients based on various prognostic parameters. In recent years, nomograms have been proven to be more accurate than traditional cancer staging systems for the prediction of malignant gastrointestinal tumors such as hepatocellular carcinoma and intrahepatic cholangiocarcinoma (23, 24).

All of the previous studies published to predict the prognosis of pCCA have only focused on the death of patients but have ignored recurrence. As a consequence, a more accurate prognostic model of individual pCCA patients can screen out the population of high-risk recurrence so that postoperative preventive adjuvant therapy can be more recommended. In particular, using a multicenter database, the object of this study was to develop and validate a prognostic model to predict recurrence-free survival (RFS) after curative pCCA resection.

## Methods

### Study Population

This is a retrospective study. Following open curative resection for newly diagnosed pCCA between January 2008 and January 2016 at three hospitals in China, patients were enrolled in a multicenter database (Southwest Hospital, Sichuan Provincial People’s Hospital, and Affiliated Hospital of Qinghai University). The diagnosis of pCCA was confirmed by postoperative histological examination. Patients with tumors emerging from the biliary confluence, right or left hepatic duct, or common hepatic duct were included in the study. The exclusion criteria were as follows: 1) recurrent pCCA; 2) neoadjuvant therapy; 3) palliative resection (R1 & R2 resection); 4) no liver resection; 5) death within 30 days after surgery; 6) missing data on important prognostic variables, including CA 19-9, maximum tumor size, macrovascular or microvascular invasion, tumor differentiation, and LN involvement; and 7) loss to follow-up. All patients underwent hepatectomy and extrahepatic bile duct resection. Regardless of whether the preoperative radiology examination suspects lymph node involvement, all patients underwent locoregional lymphadenectomy, including 8, 9, 12, and 16 stations of lymph nodes (LNs). To achieve curative resection, patients received hepatectomy-pancreaticoduodenectomy and/or revascularization when required. Patients received revascularization when the vasculature of the reserved side liver was violated. Curative resection was defined as complete resection of all microscopic and macroscopic pCCA tumors with microscopically clear resection margins in the surgical specimens. Using random assignment, 70% of patients were assigned to a training cohort, and the remaining 30% were assigned to the validation cohort. This study followed the ethical guidelines of the WMA (World Medical Association; Declaration of Helsinki). Approval for this study research was obtained from the Ethics Committee of Southwest Hospital (approval number: KY2021129). All patients provided written informed consent prior to participation in this clinical study.

### Data Collection

Clinical, laboratory, pathological and surgical variables were recorded for all patients. Clinical variables included age, sex, American Society of Anesthesiologists (ASA) score, diabetes mellitus, obesity, and preoperative drainage. Laboratory variables included alanine aminotransferase (ALT), aspartate transaminase (AST), platelets (PLT), albumin (ALB), total bilirubin (TB), international normalized ratio (INR), and carbohydrate antigen 19-9 (CA 19-9). Pathological variables included cirrhosis, maximum tumor size, macrovascular invasion, microvascular invasion, peripheral nerve invasion, tumor differentiation, 8th AJCC stage, Bismuth classification, and LN involvement. Surgical variables included perioperative blood transfusion, intraoperative blood loss, extent of hepatectomy (minor and major), and number of examined LNs (ELN).

For laboratory variables, we used the upper or lower limit of the normal values in clinical practice to divide patients into normal or high/low groups, including 40 U/L for ALT and AST, 100 ×10^9^/L for PLT, 35 g/L for ALB, 1 mg/dL for TB, and 1.25 for INR. Based on the previous studies, although 37 U/L is the upper limit of the normal value of CA199, to obtain the strongest predictive value, this study used 150 U/L as the cutoff value for CA19-9 (25, 26). Cirrhosis was confirmed by postoperative histological examination of the noncancerous resected specimen. Maximum tumor size, macrovascular invasion, microvascular invasion, peripheral nerve invasion, tumor differentiation, and LN involvement were confirmed by postoperative histological examination of the cancerous resected specimen. Tumor stage and categorization were determined according to the 8th AJCC stage and Bismuth classification (27, 28). Tumor size > 3 cm is commonly considered to be a factor leading to a poor prognosis. This study used 3 and 5 cm to divide all patients into three groups. In addition, this study divided the lymph node status into three groups: positive, negative (ELN < 4), and negative (ELN ≥ 4). Minor hepatectomy was defined as the resection of two or fewer Couinaud liver segments, and major hepatectomy was defined as the resection of three or more segments.

### Patient Follow-Up

All patients were followed up at regular intervals (approximately 1-2 months) after discharge. A standard protocol was used to evaluate the presence of pCCA recurrence, which included clinical symptoms, laboratory (tumor biomarkers and liver function), physical examinations, and radiographic images. One abdominal contrast-enhanced ultrasound (CEUS), computed tomography (CT), or magnetic resonance imaging (MRI) was performed every two months after surgery or when tumor recurrence was suspected. The presence of new lesions seen on CEUS, CT or MRI was defined as recurrence that was treated by further treatment. The primary endpoint was recurrence-free survival (RFS), and the secondary endpoint was overall survival (OS). For recurrent patients, RFS was defined as the interval from surgery to the diagnosis of tumor recurrence. For nonrecurrent patients, RFS was defined as the interval from surgery to death or last follow-up. OS was defined as the interval from surgery to death or last follow-up. The database was censored on November 15, 2020.

### Statistical Analysis

Categorical variables are expressed as numbers and percentages. The *χ^2^* test or Fisher’s exact test was used as appropriate. RFS was assessed using the Kaplan–Meier method. Univariable and multivariable analyses were performed using Cox regression with forward stepwise variable selection to identify factors to predict RFS. Variables significant at a *P* value < 0.1 in univariable analysis were entered into multivariable Cox regression analysis. The algorithm used in choosing factors for the nomogram was based on independent variables associated with RFS on multivariable Cox regression analysis to construct the nomogram model, which was formulated in R for predicting the probability of 1-, 3-, and 5-year RFS. The nomogram was subjected to 1,000 bootstrap resamples for internal validation. The performance of the nomogram in predicting survival was evaluated by calculating the area under the curve (AUC) and concordance index (C-index). To assess the fit of the nomogram, the nomogram was calibrated by comparing the predicted RFS with the observed RFS after bias correction. The clinical validity of the nomogram was evaluated by decision curve analysis (DCA), which calculated the true and false positive rates of various risk thresholds and compensated for any deficiency of ROC curves (receiver operating characteristic curves) (29). The difference in predictive performance between the nomogram and 8^th^ AJCC stage was assessed with ROC curve analysis and DCA. Based on the median nomogram score of the patients in the training cohort, all patents were divided into a low-risk group and a high-risk group. The statistical analysis was performed using SPSS 26.0 (SPSS, Chicago, IL, USA) and R software (version 3.5.1. http://www.r-project.org/). An internet browser-based calculator based on the nomogram model was programmed in JavaScript. A *P* value < 0.05 was considered to indicate a significant difference in a 2-tailed test.

## Results

### Patients and Variables

Among the 523 patients who underwent curative open resection for pCCA between January 2008 and January 2016, we excluded 15 patients who had recurrent pCCA, 30 patients who received neoadjuvant therapy, 25 patients who underwent palliative resection (R1 & R2), 26 patients who did not undergo liver resection, and 11 patients who died within 30 days after surgery. Moreover, 36 patients who had missing data on important prognostic variables and 39 patients who were lost to follow-up were also excluded. Thus, 341 patients with newly diagnosed pCCA were included in the final analytic cohort (210 male and 131 female patients), and 27.0% of patients were older than 60 years old. Among the 341 patients in the whole cohort, 239 (70.1%) patients were randomly assigned to the training cohort, and 102 (29.9%) patients were allocated to the validation cohort, as shown in **Supplementary Figure 1**. The clinical, laboratory, pathological and surgical variables among patients in the training and validation cohorts are shown in **Table 1**. The median OS and RFS times for the whole cohort of patients were 22.0 (95% CI: 18.9-25.1) and 14.0 (95% CI: 11.1-16.8) months, respectively. The 1-, 3-, and 5-year RFS rates in the whole cohort of patients were 53.4%, 25.0%, and 17.4%, respectively. The 1-, 3-, and 5-year OS rates in the whole cohort of patients were 70.9%, 32.6%, and 23.3%, respectively. The survival outcomes of the training and validation cohorts are shown in **Table 2**.

**Table 1:** Patients’ characteristics for perihilar cholangiocarcinoma.

Variables	Whole cohort (N = 341)	Training cohort (N = 239)	Validation cohort (N = 102)
Age (years), ≤ 60/> 60	249/92 (73.0/27.0)	176/63 (73.6/26.4)	73/29 (71.6/28.4)
Gender, Female/Male	131/210 (38.4/61.6)	96/143 (40.2/59.8)	35/67 (38.4/61.6)
ASA score > 2	27 (7.9)	19 (7.9)	8 (7.8)
Diabetes mellitus	31 (9.1)	20 (8.4)	11 (10.8)
Obesity	59 (17.3)	40 (16.7)	19 (18.6)
Preoperative drainage, No/Yes	230/111 (67.4/32.6)	164/75 (68.6/31.4)	66/36 (64.7/35.3)
ALT (U/L), ≤ 40/> 40	52/289 (15.2/84.8)	36/203 (15.1/84.9)	16/86 (15.7/84.3)
AST (U/L), ≤ 40/> 40	49/292 (14.4/85.6)	33/206 (13.8/86.2)	16/86 (15.7/84.3)
PLT (×10^9^/L), ≥ 100/≤ 100	325/16 (95.3/4.7)	228/11 (95.4/4.6)	97/5 (95.1/4.9)
ALB (g/L), ≥ 35/≤ 35	223/118 (65.4/34.6)	159/80 (66.5/33.5)	64/38 62.7/37.3)
TB (mg/dL), ≤ 1/> 1	69/272 (20.2/79.8)	46/193 (19.2/80.8)	23/79 (22.5/77.5)
INR, ≤ 1.25/> 1.25	293/48 (85.9/14.12)	208/31 (87.0/13.0)	85/17 (83.3/16.7)
CA 19-9 (U/L), ≤ 150/> 150	147/194 (43.1/56.9)	106/133 (44.4/55.6)	41/61 (40.2/59.8)
Cirrhosis	28 (8.2)	20 (8.4)	8 (7.8)
Maximum tumor size (cm), < 3/3-5/> 5	152/159/30 (44.6/45.6/8.8)	106/111/22 (44.4/46.4/9.2)	46/48/8 (45.1/47.1/7.8)
Macrovascular invasion, No/Yes	187/154 (54.8/45.2)	130/109 (54.4/45.6)	57/45 (55.9/44.1)
Microvascular invasion, No/Yes	285/56 (83.6/16.4)	198/41 (82.8/17.2)	87/15 (85.3/14.7)
Peripheral nerve invasion, No/Yes	216/125 (63.3/36.7)	153/86 (64.0/36.0)	63/39 (61.8/38.2)
Tumor differentiation, Well/moderate/Poor	286/55 (83.9/16.1)	201/38 (84.1/15.9)	85/17 (83.3/16.7)
8^th^ AJCC stage, I-II/III-IV	121/220 (35.5/64.5)	91/148 (38.1/61.9)	30/72 (29.4/70.6)
Bismuth classification, I-II/III-IV	71/270 (20.8/79.2)	52/187 (21.8/78.2)	19/83 (18.6/81.4)
Lymph node involvement, No (ELN > 4)/No (ELN ≤ 4)/Yes	82/128/131 (24.0/37.5/38.4)	59/90/90 (24.7/37.7/37.7)	23/38/41 (22.5/37.3/40.2)
Perioperative blood transfusion, No/Yes	115/226 (33.7/66.3)	83/156 (34.7/65.3)	32/70 (31.4/68.6)
Intraoperative blood loss (ml), ≤ 500/> 500	127/214 (37.2/62.8)	90/149 (37.7/62.3)	37/65 (36.3/63.7)
Extent of hepatectomy, Minor/Major	107/234 (31.4/68.6)	78/161 (32.6/67.4)	29/73 (28.4/71.6)

AJCC, American Joint Committee on Cancer; ALB, albumin level; ALT, alanine aminotransferase; ASA, American Society of Anesthesiologists; AST, aspartate transaminase; CA19-9, carbohydrate antigen 19-9; INR, international normalized ratio; PLT, platelets level; TB, total bilirubin.

**Table 2:** Survival outcomes for perihilar cholangiocarcinoma.

Survival outcomes	Whole cohort (N = 341)	Training cohort (N = 239)	Validation cohort (N = 102)
Period of follow-up, months*	25.7 ± 23.4	25.7 ± 22.7	26.0 ± 25.0
Recurrence during the follow-up	255 (74.8)	180 (75.3)	75 (73.5)
Death during the follow-up	231 (67.7)	163 (68.2)	68 (66.7)
OS, months**	22.0 (18.9-25.1)	23.0 (19.2-26.8)	19.0 (12.3-25.7)
1-year OS rate, %	70.9	72.6	67.1
3-year OS rate, %	32.6	32.2	33.3
5-year OS rate, %	23.3	21.9	26.4
RFS, months**	14.0 (11.1-16.8)	16.0 (12.5-19.5)	13.0 (6.5-19.5)
1-year RFS rate, %	53.4	54.8	50.1
3-year RFS rate, %	25.0	24.6	25.9
5-year RFS rate, %	17.4	15.8	21.0

*Values are mean ± standard deviation. **Values are median and 95% confidence interval.

OS, overall survival; RFS, recurrence-free survival.

### Predictors of RFS and Development of the Nomogram Model

**Figure 1:**
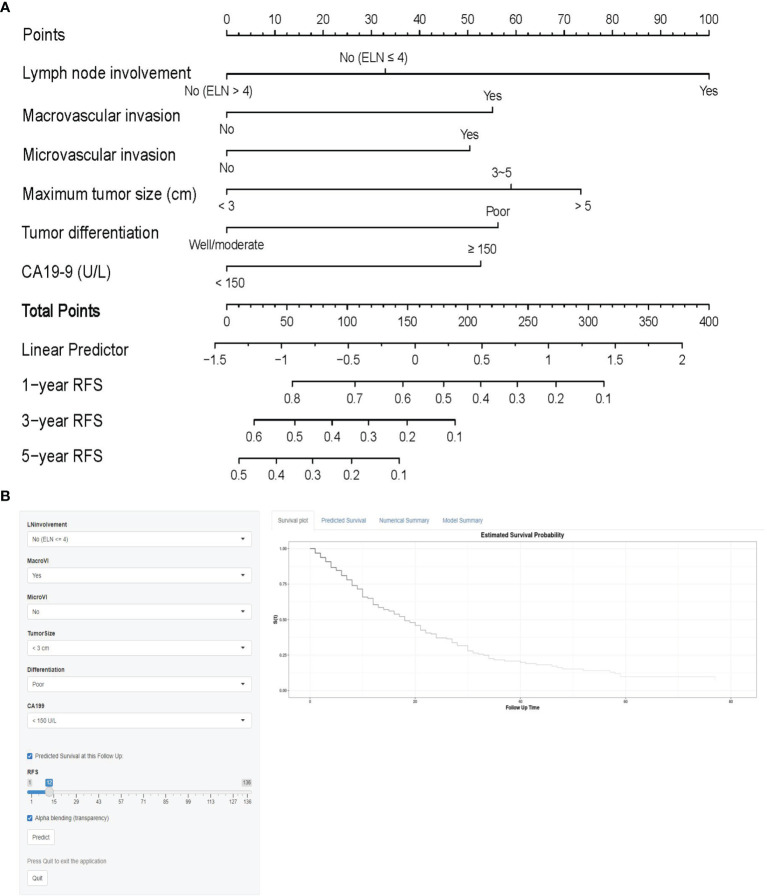
Prognostic model **(A)** and online model **(B)** for the prediction of 1-, 3-, and 5-year RFS for perihilar cholangiocarcinoma. CA19-9, carbohydrate antigen 19-9; ELN, total number of lymph nodes examined; RFS, recurrence-free survival.

**Figure 2:**
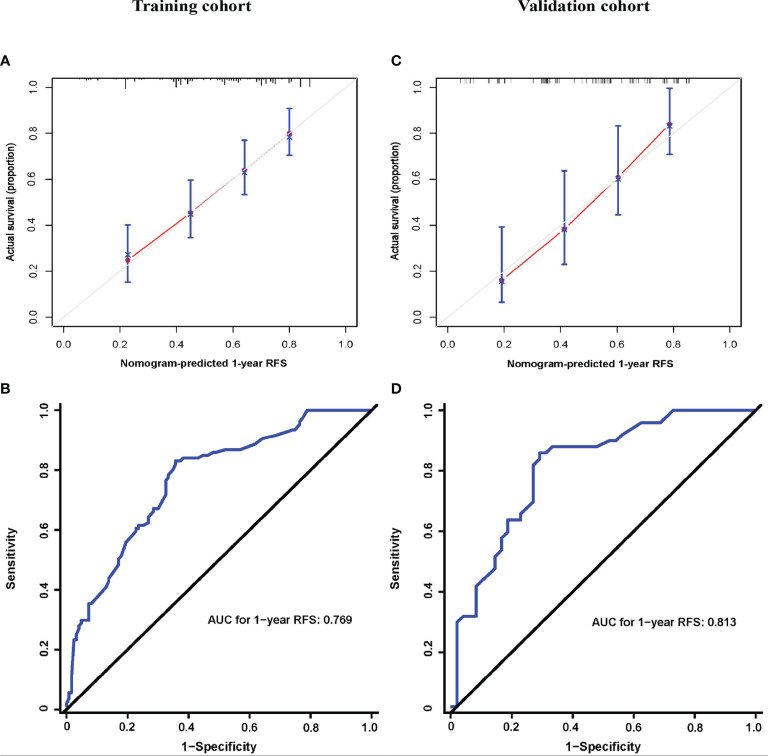
Prognostic model properties. Calibration **(A, C)** and ROC curves **(B, D)** of the prognostic model for the training **(A, B)** and validation cohorts **(C, D)**. AJCC, American Joint Committee on Cancer; AUC, area under curve; RFS, recurrence-free survival.

On univariable and multivariable Cox regression analyses, six variables were independently associated with RFS for pCCA, as shown in **Table 3**, including CA 19-9 (> 150 *vs.* ≤ 150 U/L) (HR: 1.601, 95% CI: 1.162-2.206); maximum tumor size (3~5 *vs.* < 3 cm) (HR: 1.688, 95% CI: 1.217-2.340), maximum tumor size (> 5 *vs.* < 3 cm) (HR: 1.926, 95% CI: 1.178-3.147); macrovascular invasion (yes *vs.* no) (HR 1.629, 95% CI: 1.198-2.216); microvascular invasion (yes *vs.* no) (HR: 1.566, 95% CI: 1.066-2.300); tumor differentiation (poor *vs.* well/moderate) (HR: 1.635, 95% CI: 1.082-2.470); LN involvement [no (ELN ≤ 4) *vs.* no (ELN > 4)] (HR: 1.340, 95% CI: 0.889-2.020), LN involvement [yes *vs.* no (ELN > 4)] (HR: 2.421, 95% CI: 1.605-3.652). A nomogram model that enrolled these six independent risk factors for RFS for pCCA was constructed, as shown in **Figure 1A**. Each variable was assigned a score on a point scale. By adding the scores of each variable, locating the total score on the total score table, and drawing a straight line down vertically, the probability of 1-, 3-, and 5-year RFS could be determined. In addition, the model was made *via* a free browser-based model, which is available at https://wangyeliexiantu.shinyapps.io/DynNomapp/, as shown in **Figure 1B**. The prognostic model demonstrated good calibration for risk estimation in the training cohort, as shown in **Figure 2A**. The nomogram also demonstrated good performance in predicting the probability of 1-year RFS, with an AUC of 0.769 (95% CI: 0.708–0.829) in the training cohort, as shown in **Figure 2B**.

**Table 3:** Univariable and multivariable Cox regression analyses for RFS of the training cohort.

Variables		Univariable analyses		Multivariable analyses*	
		*P*	HR (95% CI)	*P*	HR (95% CI)
Age	> 60 *vs.* ≤ 60 years	.303	1.185 (0.858-1.636)		
Gender	Male *vs.* Female	.386	0.877 (0.652-1.180)		
ASA score	> 2 *vs.* ≤ 2	.253	1.350 (0.807-2.259)		
Diabetes mellitus	Yes *vs.* No	.397	1.234 (0.758-2.010)		
Obesity	Yes *vs.* No	.995	1.001 (0.679-1.476)		
Preoperative drainage	Yes *vs.* No	.772	1.059 (0.773-1.450)		
ALT	> 40 *vs.* ≤ 40 U/L	.346	1.222 (0.805-1.856)		
AST	> 40 *vs.* ≤ 40 U/L	.583	1.131 (0.730-1.752)		
PLT	< 100 *vs.* ≥ 100 ×10^9^/L	.573	1.226 (0.603-2.494)		
ALB	< 35 *vs.* ≥ 35 g/L	.490	1.116 (0.818-1.522)		
TB	> 1 *vs.* ≤ 1 mg/dL	.712	1.074 (0.735-1.571)		
INR	> 1.25 *vs.* ≤ 1.25	.807	1.058 (0.671-1.669)		
CA 19-9	> 150 *vs.* ≤ 150 U/L	<.001	1.931 (1.426-2.616)	.004	1.601 (1.162-2.206)
Cirrhosis	Yes *vs.* No	.647	1.128 (0.674-1.885)		
Maximum tumor size	3-5 *vs.* < 3 cm	<.001	2.154 (1.566-2.961)	.002	1.688 (1.217-2.340)
	> 5 vs. < 3 cm	.013	1.840 (1.135-2.982)	.009	1.926 (1.178-3.147)
Macrovascular invasion	Yes *vs.* No	<.001	1.948 (1.445-2.625)	.002	1.629 (1.198-2.216)
Microvascular invasion	Yes *vs.* No	.002	1.836 (1.261-2.672)	.022	1.566 (1.066-2.300)
Peripheral nerve invasion	Yes *vs.* No	.748	1.051 (0.776-1.424)		
Tumor differentiation	Poor *vs.* Well/moderate	.009	1.691 (1.138-2.514)	.020	1.635 (1.082-2.470)
Lymph node involvement	No (ELN ≤ 4) *vs.* No (ELN > 4)	.066	1.460 (0.975-2.186)	.162	1.340 (0.889-2.020)
	Yes *vs.* No (ELN > 4)	<.001	2.713 (1.818-4.049)	<.001	2.421 (1.605-3.652)
Perioperative blood transfusion	Yes *vs.* No	.528	1.106 (0.809-1.510)		
Intraoperative blood loss (ml)	> 500 *vs.* ≤ 500 ml	.358	1.154 (0.850-1.566)		
Extent of hepatectomy	Major *vs.* Minor	.518	1.108 (0.811-1.514)		

*Those variables found significant at P <.100 in univariable analyses were entered into multivariable Cox regression analyses.

ALB, albumin level; ALT, alanine aminotransferase; ASA, American Society of Anesthesiologists; AST, aspartate transaminase; CA19-9, carbohydrate antigen 19-9; CI, confidence interval; HR, hazard ratio; INR, international normalized ratio; PLT, platelets level; RFS, recurrence-free survival; TB, total bilirubin.

### Validation and Clinical Applicability

The prognostic model calibration demonstrated similarly a good fit in the validation cohort, and the prediction for the probability of 1-year RFS agreed with actual observations, as shown in **Figure 2C**. Meanwhile, the nomogram performed similarly well when applied to the validation cohort to predict the probability of 1-year RFS for pCCA, with an AUC of 0.813 (95% CI: 0.728–0.898), as shown in **Figure 2D**.

**Figure 3:**
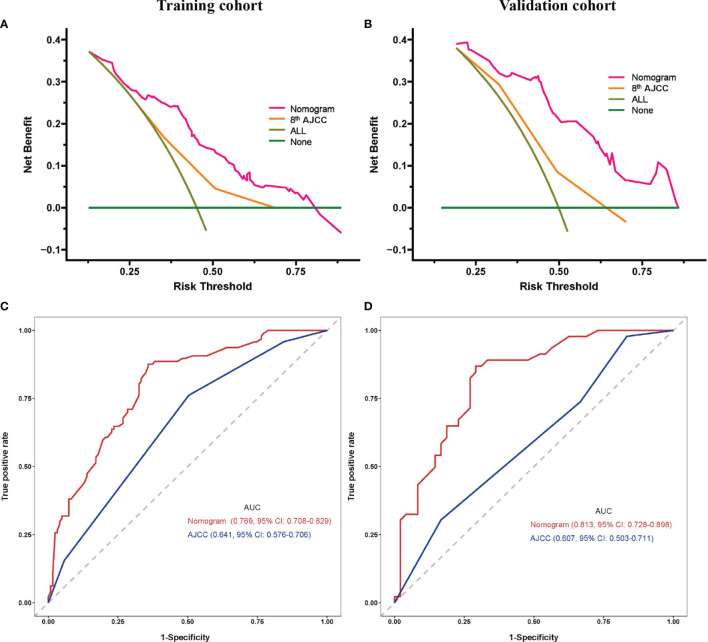
Prognostic model comparisons. Decision curve analysis **(A, C)** and ROC curves **(B, D)** of the prognostic model and 8th AJCC stage for the training **(A, B)** and validation cohorts **(C, D)**. AJCC, American Joint Committee on Cancer; AUC, area under curve; CI, confidence interval.

DCA demonstrated that using this prognostic model to predict the probability of 1-year RFS provided more benefit than the 8th AJCC stage in both the training and validation cohorts, as shown in **Figure 3A**, **B**, respectively. In addition, the nomogram model had a higher AUC than the 8th AJCC stage for predicting 1-year RFS in the training and validation cohorts, as shown in **Figure 3C**, **D**, respectively. In the training cohort, the discriminatory ability of the prognostic model had a C-index of 0.723 (95% CI: 0.684-0.762), which was superior to the 8th AJCC stage (C-index: 0.641, 95% CI: 0.576-0.706). In the validation cohort, the discriminatory ability of the prognostic model had a C-index of 0.743 (95% CI: 0.688-0.798), which was superior to the 8th AJCC stage (C-index: 0.607, 95% CI: 0.503-0.711). Notably, the prognostic model also performed better than the 8th AJCC stage for the prediction of 1-year OS in both the training and validation cohorts, as shown in **Table 4**.

**Table 4:** Comparison of the prognostic accuracies for 1-year RFS and OS of the nomogram and the 8th AJCC stage.

		Nomogram	8^th^ AJCC stage	*P*
		Training cohort		
RFS	C-index (95% CI)	0.723 (0.684-0.762)	0.641 (0.576-0.706)	< 0.001
OS	C-index (95% CI)	0.764 (0.727-0.801)	0.617 (0.580-0.654)	< 0.001
		Validation cohort		
RFS	C-index (95% CI)	0.743 (0.688-0.798)	0.607 (0.503-0.711)	< 0.001
OS	C-index (95% CI)	0.720 (0.663-0.777)	0.541 (0.470-0.612)	< 0.001

AJCC, American Joint Committee on Cancer; C-index, concordance index; OS, overall survival; RFS, recurrence-free survival.

### Risk Group Stratification Based on the Nomogram Score

**Figure 4:**
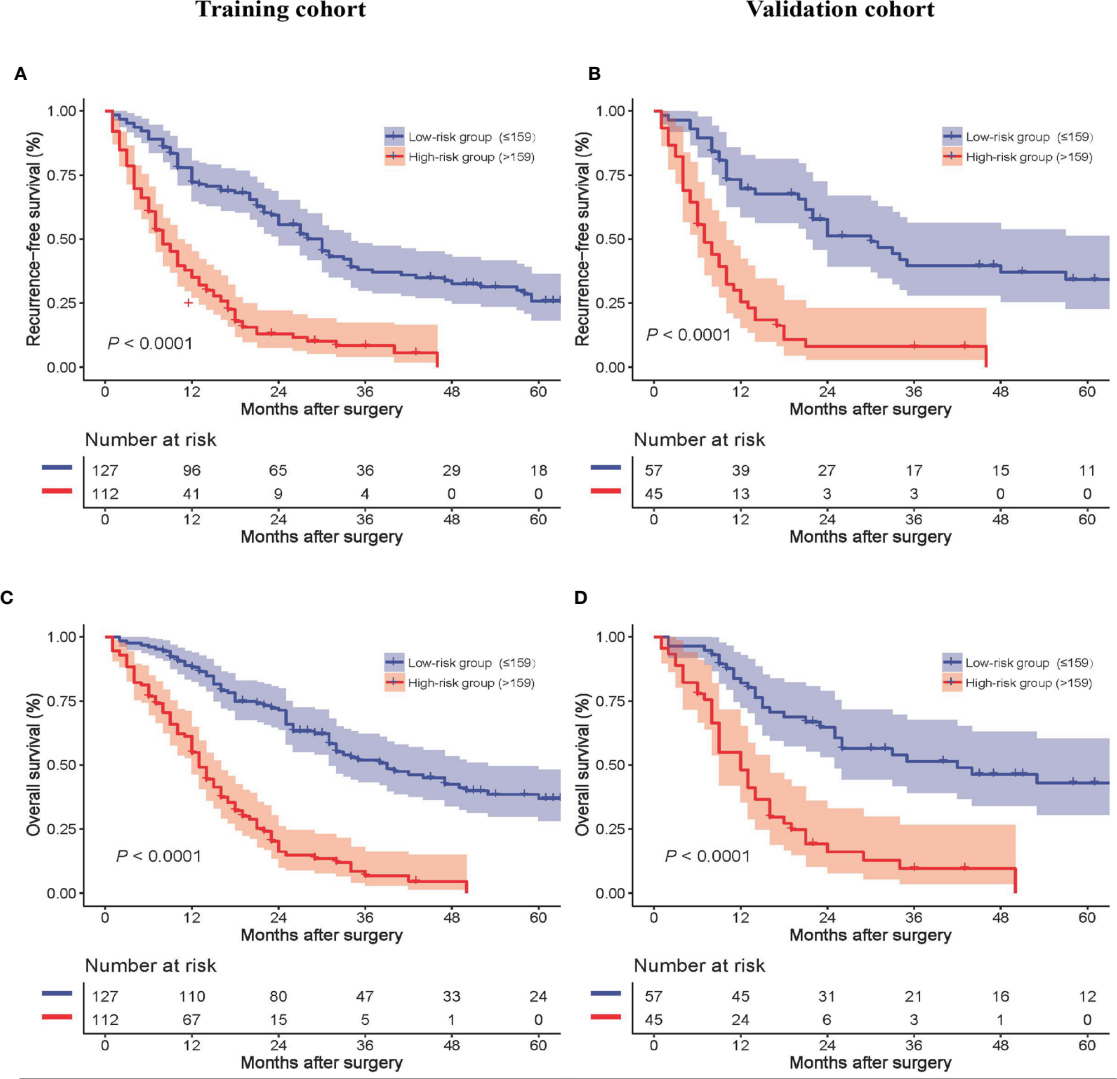
Recurrence-free survival of all patients between the low- and high-risk groups in the training **(A)** and validation cohorts **(B)**. Overall survival of all patients between the low- and high-risk groups in the training **(C)** and validation cohorts **(D)**.

The median model score of the training cohort, 159, effectively distinguished populations of different recurrence risks in the training and validation cohorts. Patients with a model score > 159 had a high risk of recurrence, and patients with a model score ≤ 159 had a low risk of recurrence. The formula for calculating the model score is shown in **Supplementary Table 1**. The RFS of high-risk patients was inferior to that of low-risk patients in both the training and validation cohorts, as shown in **Figures 4A**, **B**, respectively. In addition, the OS of high-risk patients was inferior to that of low-risk patients in both the training and validation cohorts, as shown in **Figures 4C**, **D**, respectively.

## Discussion

Traditionally, Bismuth-Corlette, Memorial Sloan-Kettering Cancer Center, and Blumgart staging systems are mostly used to evaluate the respectability of pCCA according to the tumor location in the biliary tree, portal vein invasion, and liver lobe atrophy status (30). According to the abovementioned stage, clinical surgeons are able to choose the most suitable surgical methods (30). After curative surgery, tumor recurrence is the main cause of death in pCCA patients, so clinicians urgently need a tool that can accurately predict recurrence. An effective prediction of the long-term oncologic prognosis can not only be used to refer to the frequency and duration of follow-up needed but can also provide a basis for further adjuvant treatment after surgery. However, little attention has been given to stage when evaluating the patient’s prognosis after surgery. The AJCC TNM is a widely used staging system that can not only guide the preoperative treatment plan but also predict the postoperative prognosis of patients (7). Unfortunately, the AJCC TNM staging only includes the indicators of the tumor itself, so it is not accurate enough in predicting long-term survival (7). The nomogram is a visual and simple model that is able to predict the survival outcome in various tumors and has been widely used in clinical practice due to its feasibility and accuracy (31–33). Thus, in this study, an online prognostic model was developed and validated to predict RFS after curative resection of pCCA. The model was presented as a nomogram and an online model, and the analysis results showed that the model had excellent predictive performance, with a C-index of 0.723 in the training cohort and 0.743 in the validation cohort. Calibration was also excellent in both the training and validation cohorts. This prognostic model clearly outperformed the 8th AJCC TNM staging system.

This prognostic model was based on six independent risk factors that are present in the histology and serum tumor biomarker report of every resected pCCA, including LN involvement and count, macro- and microvascular invasion, maximum tumor size, tumor differentiation, and CA 19-9. LN involvement is commonly considered to be an independent predictor for poorer oncologic prognosis in pCCA patients (34). Notably, when positive LNs are not found, the examination of less than four LNs can cause understaging and is independently associated with poor prognosis (13). For tumors of the biliary system, lymphatic metastasis is a very important dissemination method for metastasis. Therefore, we believe that pCCA patients, regardless of whether imaging suggests metastasis, should routinely undergo lymphatic dissection. This is not only an essential step for radical treatment but also an important factor in clarifying the prognosis of patients. Tumor size was confirmed to be associated with the long-term survival of pCCA patients. DeOliveira et al. emphasized that patients with tumors larger than 3 cm have a poorer prognosis than those with smaller tumors (30). In addition, a larger tumor may indicate a poorer prognosis. For example, tumor size > 5 cm was revealed to be independently associated with poor long-term survival of pCCA (17). This may be because the location of pCCA is extremely special and often does not have a complete envelope. Therefore, as the size of the pCCA tumor continues to increase, the probability of it invading the hepatic artery and portal vein may also increase. The scope of the tumor is increasing; at the cytological level, the possibility of early metastasis is increasing. Even if the margins are negative or the tumor is not visible to the eye, the possibility of complete elimination of tumor cells is reduced. Traditionally, it was believed that portal vein invasion had no effect on the long-term prognosis and could only determine the respectability of the tumor. However, recent research indicates that portal vein invasion was independently associated with worse OS than portal vein invasion (19). Although hepatic artery invasion commonly did not have an association with the resectability of pCCA, it had a significant effect on the poor prognosis of the patients. Branch or main hepatic artery invasion patients showed a poor OS compared to those without hepatic artery invasion due to the promotion of pCCA metastasis by hepatic artery invasion (35). Furthermore, we believe that, for pCCA, the tissue in which the tumor invades is related to the location of the tumor’s initial growth and not directly related to the degree of malignancy of the tumor. Invasion of the hepatic artery or portal vein does not imply a difference in malignancy. As long as R0 resection can be achieved, the prognosis of patients will be prolonged. Therefore, we unified portal vein invasion and hepatic artery invasion as macrovascular invasion. CA19-9 has been widely used as a diagnostic or prognostic biomarker for several gastrointestinal cancers, including cholangiocarcinoma, gastric cancer, and colorectal cancer (36–38). pCCA patients with preoperative CA19-9 levels < 150 U/ml showed better long-term survival outcomes than those with higher CA19-9 levels (26). Moreover, a study found a negative association between preoperative serum CA19-9 levels and the survival time of pCCA patients (19). However, the underlying mechanisms for the aberrant serum CA19-9 levels in pCCA patients are still unknown. In addition, tumor differentiation and microvascular invasion were both demonstrated to be independent predictive factors and to have a strong impact on the oncologic prognosis of resected pCCA (39–41).

The model can screen out high-risk recurrence patients (score > 159), guide decision-making for postoperative preventive adjuvant therapy, and help to decrease the incidence of recurrence, thereby prolonging the survival time of patients. At present, the role of adjuvant therapy in patients with resected pCCA is poorly defined, and there is a lack of data from phase III randomized controlled trials (42, 43). Therefore, we believe that for patients with a low risk of recurrence, follow-up should be strengthened initially instead of providing adjuvant therapy immediately. At the same time, we need to find the reasons for the low-risk recurrence of factors other than our model, such as whether these patients have already received postoperative chemotherapy. Several retrospective studies have suggested that adjuvant chemoradiation may improve long-term survival and local control, although distant metastases are still the most common mode of failure (44–47). Other researchers have suggested that adjuvant chemoradiation may have significant benefits only in patients with T3 or T4 tumors or those with a high risk of locoregional recurrence (positive margin or LN involvement) (46, 48, 49). In a systematic review and meta-analysis, Horgan et al. revealed an associated improvement in survival time (although nonsignificant) with adjuvant therapy compared with resection alone (50). Another systematic review and meta-analysis of 21 clinical trials indicated a significantly higher 5-year OS with postoperative adjuvant therapy in patients with extrahepatic cholangiocarcinoma (51). In addition, targeted therapy has made some progress in controlling recurrence. A phase III study including 185 patients with advanced *IDH1*-mutant cholangiocarcinoma caused significant improvement in progression-free survival (median 2.7 months *vs.* 1.4 months; HR: 0.37, *P* < 0.001) when treated with an *IDH1* inhibitor named ivosidenib compared to placebo (52). Therefore, we believe that patients with a high risk of recurrence should be screened out, and while follow-up is strengthened, postoperative adjuvant therapy should be recommended.

The first published prognostic model for pCCA is a risk score calculated with age, margin status, T stage, and adjuvant chemoradiation (53). This was flawed because it included only 96 patients and lacked data on important prognostic indicators, including lymph node status. Recently, Koerkamp et al. proposed a prognostic model for pCCA patients (18). In their model, three indicators, including LN status and count, differentiation, and margin status, were independent risk factors that affect disease-specific survival in patients with pCCA after surgery (18). Although the C-index of this model was 0.73, which showed a high predictive value for the oncologic prognosis of pCCA, our team thinks that it still has some limitations. For example, data from Asian populations are lacking, as well as serum tumor biomarkers such as CA19-9. Zhang et al. used the database from Surveillance, Epidemiology and End Results (SEER) to develop a more detailed tumor size model to predict the cancer-specific survival of pCCA, which was validated by Asian populations (20). However, the C-index of this model was only 0.626, and it also lacked serum tumor biomarkers, such as CA 19-9. Therefore, when our model was developed, our team specifically considered the importance of CA 19-9 to prognosis and added this parameter to our model. In addition, the data used to develop the abovementioned model were all from the SEER database or a single-center Western database because of the lack of data modeling in Eastern populations. In addition, none of the above models predict the recurrence of patients. Based on the multicenter Eastern database, we developed and validated an online prognostic model containing tumor biomarkers with excellent performance in predicting RFS.

This study has several limitations. First, this model lacked western external validation. We tried to use the SEER database for validation, but the SEER database lacked information on preoperative serum tumor biomarkers. Cooperating with other institutions for external validation is what we should continue to do. Second, 1 to 3 of the patients in this study had fewer than four LNs examined, and these patients were potentially understaged due to insufficient LN evaluation, which could rule out LN metastasis. Collecting at least four LNs has been essential. Previous research indicated that LN-negative patients had poorer long-term survival if fewer than four LNs were examined (14). However, although lymphadenectomy is a standard part of curative intent resection, most surgeries still have a high percentage of patients with fewer than four LNs examined. Thus, in our study, the LN status and count were all collected and added to the model to largely resolve the limitation. Third, only patients with R0 resection were included. Determining whether patients with R1 or R2 resection are suitable for this model requires more research. Fourth, this study lacked data for postoperative adjuvant therapy. The patients included in this study were recruited between 2008 and 2016. During this time, because there is a dearth of evidence from phase III RCTs, the usefulness of adjuvant chemotherapy or chemo-radiation therapy in patients with resected pCCA is unclear (42, 43). Therefore, we did not have a detailed record of data for postoperative adjuvant chemotherapy. However, more evidence proves that postoperative adjuvant chemotherapy may be beneficial for pCCA patients. We will perform more detailed records for adjuvant therapy in future studies.

## Conclusion

Using a multicenter database, a prognostic model was developed and validated that can effectively predict 1-year RFS and screen out patients at high risk for recurrence (score > 159). Our research revealed that this model has significantly better predictive performance and clinical applicability than the 8th AJCC TNM staging system. The model is available as a simple and visual calculator *via* the web, making it more convenient for clinicians to apply. Further prospective, large-scale, external validation in Western cohorts is warranted.

## Data Availability Statement

The raw data supporting the conclusions of this article will be made available by the authors, without undue reservation.

## Ethics Statement

The studies involving human participants were reviewed and approved by Ethics Committee of Southwest Hospital. The patients/participants provided their written informed consent to participate in this study. Written informed consent was obtained from the individual(s) for the publication of any potentially identifiable images or data included in this article.

## Author Contributions

Conception: H-SD, Z-YC. Study design: Z-PL, W-YC, LX, H-SD, Z-YC. Administrative support: H-SD, Z-YC. Data collection and acquisition: Z-PL, LX, YP, S-YZ, JB, YJ. Data analysis: Z-PL, W-YC, Z-RW, LX, Y-QZ, H-SD, Z-YC. Manuscript preparation: Z-PL, W-YC, Z-RW, YP, S-YZ, H-SD, Z-YC. Critical revision: Y-QZ, H-SD, Z-YC. Final approval of manuscript: All authors. Z-PL and W-YC contributed equally to this work. All authors contributed to the article and approved the submitted version.

## Funding

This work was supported in part by the National Natural Science Foundation of China (No. 81874211) and Talent Training Program of Army Medical University (No. XZ-2019-505-014).

## Conflict of Interest

The authors declare that the research was conducted in the absence of any commercial or financial relationships that could be construed as a potential conflict of interest.

## Publisher’s Note

All claims expressed in this article are solely those of the authors and do not necessarily represent those of their affiliated organizations, or those of the publisher, the editors and the reviewers. Any product that may be evaluated in this article, or claim that may be made by its manufacturer, is not guaranteed or endorsed by the publisher.

## Supplementary Material

Supplementary Figure 1 Flow chart of patient inclusion. pCCA, perihilar cholangiocarcinoma.

Supplementary Table 1 The corresponding score in our prediction model. ELN, examined lymph nodes; Carbohydrate antigen 19-9. Model score = Lymph node involvement + Maximum tumor size + Macrovascular invasion + Microvascular invasion + Tumor differentiation + CA 19-9.

## Abbreviations

ALB, albumin; ALT, alanine aminotranferase; AJCC, American Joint Committee on Cancer; ASA, American Society of Anesthesiologists; AST, aspartate transaminase; AUC, area under the curve; CA19-9, Carbohydrate antigen 19-9; CCA, cholangiocarcinoma; CI, confidence interval; C-index, Concordance index; CT, computed tomography; DCA, decision curve analysis; ELN, examined lymph nodes; HR, hazard ratio; INR, international normalized ratio; LN, lymph node; MRI, magnetic resonance imaging; OS, overall survival; pCCA, perihilar cholangiocarcinoma; RFS, recurrence-free survival; PLT, platelet; ROC, receiver operating characteristic; SEER, epidemiology and end results; TB, total bilirubin; WMA, World Medical Association.
