# Supplementary material for: Development and Validation of a Prognostic Model to Predict Recurrence-Free Survival After Curative Resection for Perihilar Cholangiocarcinoma: A Multicenter Study
Source: Front Oncol. 2022 Apr 21;12:849053. doi: 10.3389/fonc.2022.849053 (PMC9071302; doi:10.3389/fonc.2022.849053)
Supplement: Supplementary Figure 1 — Flow chart of patient inclusion. pCCA, perihilar cholangiocarcinoma. [file DataSheet_1.docx]

**Supplement** **Figure 1.** Flow chart of patient inclusion. pCCA, perihilar cholangiocarcinoma.

**

**
