# Supplementary material for: Development and Validation of a Prognostic Model to Predict Recurrence-Free Survival After Curative Resection for Perihilar Cholangiocarcinoma: A Multicenter Study
Source: Front Oncol. 2022 Apr 21;12:849053. doi: 10.3389/fonc.2022.849053 (PMC9071302; doi:10.3389/fonc.2022.849053)
Supplement: Supplementary Table 1 — The corresponding score in our prediction model. ELN, examined lymph nodes; Carbohydrate antigen 19-9. Model score = Lymph node involvement + Maximum tumor size + Macrovascular invasion + Microvascular invasion + Tumor differentiation + CA 19-9. [file Table_1.docx]

**Supplementary** **Table 1.** The corresponding score in our prediction model. ELN, examined lymph nodes; Carbohydrate antigen 19-9.

| **Variables** | **Assignment** | **Categories** | **Points** |
| --- | --- | --- | --- |
| Lymph node involvement | 1 | No (ELN > 4) | 0 |
|  | 2 | No (ELN ≤ 4) | 33 |
|  | 3 | Yes | 100 |
| Maximum tumor size (cm) | 1 | < 3 | 0 |
|  | 2 | 3-5 | 59 |
|  | 3 | > 5 | 73 |
| Macrovascular invasion | 0 | No | 0 |
|  | 1 | Yes | 55 |
| Microvascular invasion | 0 | No | 0 |
|  | 1 | Yes | 50 |
| Tumor differentiation | 0 | Well/moderate | 0 |
|  | 1 | Poor | 56 |
| CA 19-9 (U/L) | 0 | ≤ 150 | 0 |
|  | 1 | > 150 | 53 |

Model score = Lymph node involvement + Maximum tumor size + Macrovascular invasion + Microvascular invasion + Tumor differentiation + CA 19-9.
